# Galectin-3 level predicts response to ablation and outcomes in patients with persistent atrial fibrillation and systolic heart failure

**DOI:** 10.1371/journal.pone.0201517

**Published:** 2018-08-01

**Authors:** Nicolas Clementy, Bruno Garcia, Clémentine André, Arnaud Bisson, Nazih Benhenda, Bertrand Pierre, Anne Bernard, Laurent Fauchier, Eric Piver, Dominique Babuty

**Affiliations:** 1 Cardiology Department, Trousseau Hospital, University of Tours, Tours, France; 2 Biochemistry Department, Trousseau Hospital, University of Tours, Tours, France; Universita degli Studi di Roma La Sapienza, ITALY

## Abstract

**Introduction:**

Mechanisms of maintenance of both atrial fibrillation and structural left ventricular disease are known to include fibrosis. Galectin-3, a biomarker of fibrosis, is elevated both in patients with heart failure and persistent atrial fibrillation. We sought to find whether galectin-3 has a prognostic value in patients with heart failure and a reduced left ventricular ejection fraction undergoing ablation of persistent atrial fibrillation.

**Methods:**

Serum concentrations of galectin-3 were determined in a consecutive series of patients with an ejection fraction ≤40%, addressed for ablation of persistent atrial fibrillation. Responders to ablation were patients in sinus rhythm and with an ejection fraction ≥50% at 6 months. A combined endpoint of heart failure hospitalization, transplantation and/or death was used at 12 months.

**Results:**

Seventy-five patients were included (81% male, age 63±10 years, ejection fraction 34±7%, galectin-3 21±12 ng/mL). During follow-up, eight patients were hospitalized for decompensated heart failure, 1 underwent heart transplantation, and 4 died; 50 patients were considered as responders to ablation. After adjustment, galectin-3 level independently predicted both 6-month absence of response to ablation (OR = 0.89 per unit increase, p = 0.002). Patients with galectin-3 levels <26 had a 95% 1-year event-free survival versus 46% in patients with galectin-3 ≥26 ng/mL (p<0.0001).

**Conclusions:**

Galectin-3 levels independently predict outcomes in patients with reduced left ventricular systolic function addressed for ablation of persistent AF, and may be of interest in defining the therapeutic strategy in this population.

## Introduction

Ablation for persistent atrial fibrillation (AF) can be recommended in symptomatic patients, especially those with previous decompensated heart failure (HF) [1]. Patients who present with AF and left ventricular (LV) dysfunction may be: (1) patients with a true underlying structural heart disease associated with AF; (2) patients with no structural heart disease with a reversible arrhythmia-induced cardiomyopathy (AIC); (3) patients with a structural heart disease aggravated by AIC [2]. The diagnosis of AIC is retrospective, i.e. after partial or complete recovery of LV ejection fraction (LVEF) following restoration of sustained sinus rhythm, and it may thus be not fully determined in patients with recurrent AF.

It is still unclear how to recognize patients with reduced LVEF who will actually benefit from ablation of persistent AF: (1) even after restoration of a sustained sinus rhythm, patients with a severe cardiomyopathy will not recover; (2) sustained sinus rhythm is unlikely to be restored in patients with a true AIC and severe AF-induced atrial remodeling, i.e. fibrotic atrial cardiomyopathy, who will thus be unlikely to recover normal LV function, even after multiple extensive ablation procedures [3,4].

We have previously shown that galectin-3, a member of the ß-galactoside-binding lectin family, which plays a role in promoting atrial fibrosis in patients with AF, displays higher levels in patients with more extensive atrial remodeling [5]. Galectin-3 has been associated with new onset AF [6,7]. The prognosis for patients with higher galectin-3 levels is also worse after AF ablation [8,9]. Finally, galectin-3 levels are known to be higher in HF patients with structural heart disease [10].

We thus hypothesized that lower galectin-3 levels, reflecting a smaller fibrotic substrate area within atrial and/or ventricular chambers, could identify better candidates for ablation, so-called 'responders to ablation', i.e. persistent AF patients with a reduced LVEF who partially or completely recover after a sustained restoration of sinus rhythm.

## Materials and methods

### Inclusion

Consecutive patients ≥18 years old with symptomatic persistent AF referred to our department for ablation between January 2013 and December 2015 were analyzed. Patients with the association of symptomatic HF and an LVEF ≤40% were included. Patients with a prior ablation for AF and those in sinus rhythm at the time of baseline echocardiographic evaluation were excluded. Persistent AF was defined as continuous AF episodes sustained >7 days. Collected clinical data included symptoms and history of arrhythmia, presence of risk factors, past and current medications. Transthoracic echocardiography was systematically performed before ablation.

All methods, including the ablation procedure, were carried out in accordance with the current guidelines, and conformed to the ethical guidelines of the 1975 Declaration of Helsinki. The ethics committee for human research of the University Hospital Center of Tours (France) approved the study protocol. All patients signed informed consent before inclusion.

### Galectin-3

During the early stage of the AF ablation procedure, a blood sample was collected peripherally through the femoral vein sheath to determine anticoagulation time. Measurement of serum galectin-3 level was performed on residual samples.

The galectin-3 level was determined using the VIDAS Galectin-3 kit (bioMérieux, Marcy-l'Etoile, France), an automated quantitative test. The kit's measuring range is 3.3–100 ng/mL. The assay principle is a one-step immunoassay sandwich method with final fluorescent detection, and has already been validated in heart failure patients [11].

### Ablation

All procedures were performed under general anesthesia, with the objective for anticoagulation time set at 300 seconds. A 4-millimeter irrigated-tip catheter was used in all patients to deliver radiofrequency energy. After transeptal puncture, antral pulmonary vein isolation was performed in all patients. A bidirectional block was confirmed in all veins using a circular mapping catheter. In patients still showing arrhythmia at that stage a stepwise approach with the aim of reestablishing sinus rhythm was performed sequentially: anterior roof and mitral isthmus lines were obtained (endocardially, and epicardially through the coronary sinus, when necessary), complex fractionated atrial electrograms were mapped and targeted [12]. Stable atrial tachycardias were systematically mapped and ablated. When return to sinus rhythm was obtained, either through ablation, or by electrical cardioversion at the end of procedure, a bidirectional block was confirmed on all performed lines.

### Follow-up

All patients were monitored for 12 months. Recurrence was defined as ≥1 documented sustained episode (≥30 seconds) of any atrial arrhythmia, symptomatic or not, on any electrocardiogram or Holter monitoring strip (scheduled or additional), after a single ablation procedure and a 3-month blanking period. During the blanking period, antiarrhythmic drugs were continued in most of the patients, and a cardioversion was performed in the event of persistent recurrence. At the end of the blanking period, antiarrhythmic drugs were systematically discontinued in all patients. A clinical examination, a resting ECG and a 5-day Holter monitoring were performed at 3, 6, and 12 months of follow-up. A transthorcacic echocardiography was performed at baseline and 6-month follow-up. Echocardiographic measurements were performed blindly by a single experienced operator, using specific software (EchoPAC, GE Healthcare). Left ventricular volumes and ejection fraction were calculated using the biplane Simpson method. Mean heart rate during echocardiography was calculated by averaging 10 successive RR intervals.

### Outcomes

At 12 months, a composite criterion was used as primary hard endpoint: freedom from cardiac death, from heart transplantation and from HF hospitalization.

As a secondary endpoint, responders to ablation were defined as being those patients fulfilling all the following criteria at 6 months: (1) alive, (2) in sinus rhythm, and (3) with an LVEF ≥50%.

### Statistical analyses

Analyses were performed using JMP software version 9.0 (SAS Institute Inc., Cary, NC, USA). Numeric data were expressed as mean ± standard deviation (95% confidence interval). Parametric tests were used for comparison between groups. A logistic regression model and a Cox model were used to assess the factors independently associated with outcomes. The main confounding factors described in the literature were tested in univariable analysis [13,14]. Different multivariable models were studied: a model using all parameters significantly associated with outcomes in univariate analysis, and a model limited to 3 parameters considering the rather small number of events. Analyses by receiver operating characteristic curves were performed to assess the accuracy of galectin-3 at predicting response and outcomes, and obtain optimal cutoff values. Survival curves were obtained using the Kaplan Meier method. A p-value ≤0.05 was considered significant.

## Results

### Population

Out of 191 consecutive patients who had undergone a first ablation procedure for persistent AF, 75 patients (39%) with an LVEF ≤40% in AF were included in the study. Characteristics are reported in Table 1.

**Table 1:** Characteristics for all patients and for responders and non-responders at baseline and during follow-up.

	All Patients (N = 75)	Responders (N = 50)	Non-Responders (N = 25)	p*
**Baseline**				
Male gender (%)	61 (81)	39 (78)	22 (88)	0.28
Age (years)	63 ±10	62 ±11	66 ±10	0.22
History of cardiomyopathy (%)	43 (57)	23 (45)	20 (83)	**0.001**
Ischemic heart disease	16 (21)	4 (8)	12 (48)	**<0.0001**
Non ischemic cardiomyopathy	20 (27)	13 (25)	7 (29)	0.85
Hypertrophic cardiomyopathy	3 (4)	2 (4)	1 (4)	-
Implantable cardioverter-defibrillator	12 (16)	3 (6)	9 (38)	**0.0008**
Hypertension (%)	42 (56)	26 (52)	16 (64)	0.32
Diabetes mellitus (%)	19 (25)	10 (20)	9 (37)	0.14
Transient ischemic attack or stroke (%)	2 (3)	1 (2)	1 (4)	0.61
Vascular disease (%)	20 (27)	8 (16)	12 (50)	**0.003**
Renal failure (%)	26 (35)	13 (26)	13 (52)	**0.03**
CHA_2_DS_2-_VASC Score: 1 / 2 / 3 / >3 (%)	16 / 20 / 25 / 39	22 / 25 / 22 / 31	4 / 8 / 33 / 55	0.05
Renin-angiotensin-aldosterone inhibitor (%)	61 (81)	40 (80)	21 (84)	0.68
Betablocker (%)	75 (100)	50 (100)	25 (100)	-
Amiodarone (%)∫	68 (91)	45 (90)	23 (92)	0.78
Mean heart rate (bpm)	90 ±20	88 ±20	93 ±20	0.32
Left atrial volume (mL/m^2^)	48 ±16	49 ±16	47 ±15	0.70
Indexed LV end-diastolic diameter (mm/m^2^)	27 ±5	26 ±4	30 ±6	**0.02**
LVEF (%)	34 ±7	35 ±6	31 ±8	0.06
Galectin-3 (ng/mL)	21.4 ±12.4	17.9 ±5.2	28.4 ±18.4	**0.002**
BNP (ng/L)	445 ±650	317 ±479	700 ±857	**0.004**
**6-month (N = 71)**				
Sinus rhythm (%)	59 (79)	50 (100)	9 (43)	**<0.0001**
LVEF (%)	51 ±12	57 ±7	39 ±11	**<0.0001**
ΔLVEF (%)	17 ±11	+22 ±8	+8 ±10	**<0.0001**
**12-month**				
HF hospitalization	8 (11)	1 (2)	7 (28)	**0.0006**
Heart transplantation (%)	1 (1)	0	1 (4)	0.15
Death (%)	4 (5)	0	4 (16)	**0.004**

ΔLVEF, variation of LVEF between baseline and 6-month follow-up.

* Comparison between responders and non-responders.

∫ Amiodarone was systematically discontinued at 3 months (blanking period).

### Outcomes

No patient was lost during the 12-month follow-up period. The following events occurred: at 6 months, 16 patients had AF recurrence (persistent type for all); at 12 months, 8 patients were hospitalized for congestive HF after a median follow-up of 73 days (interquartile range 181), 1 patient underwent heart transplantation after 229 days, and 4 patients died, 3 from sudden cardiac death (SCD) and 1 from end-stage HF.

Of the 59 patients without recurrence who underwent at 6 months an echocardiographic evaluation of left ventricular function in sinus rhythm, 50 (85%) were considered responders to ablation according to the definition, and were thus diagnosed with AIC. Responders had a higher body mass index, a thinner LV end-diastolic diameter, and were less likely to have a history of ischemic heart disease or to be implanted with a cardioverter-defibrillator (Table 1). There was no significant difference for the mean heart rate in AF during echocardiography at baseline between responders and non-responders (p = 0.32).

**Fig 1:**
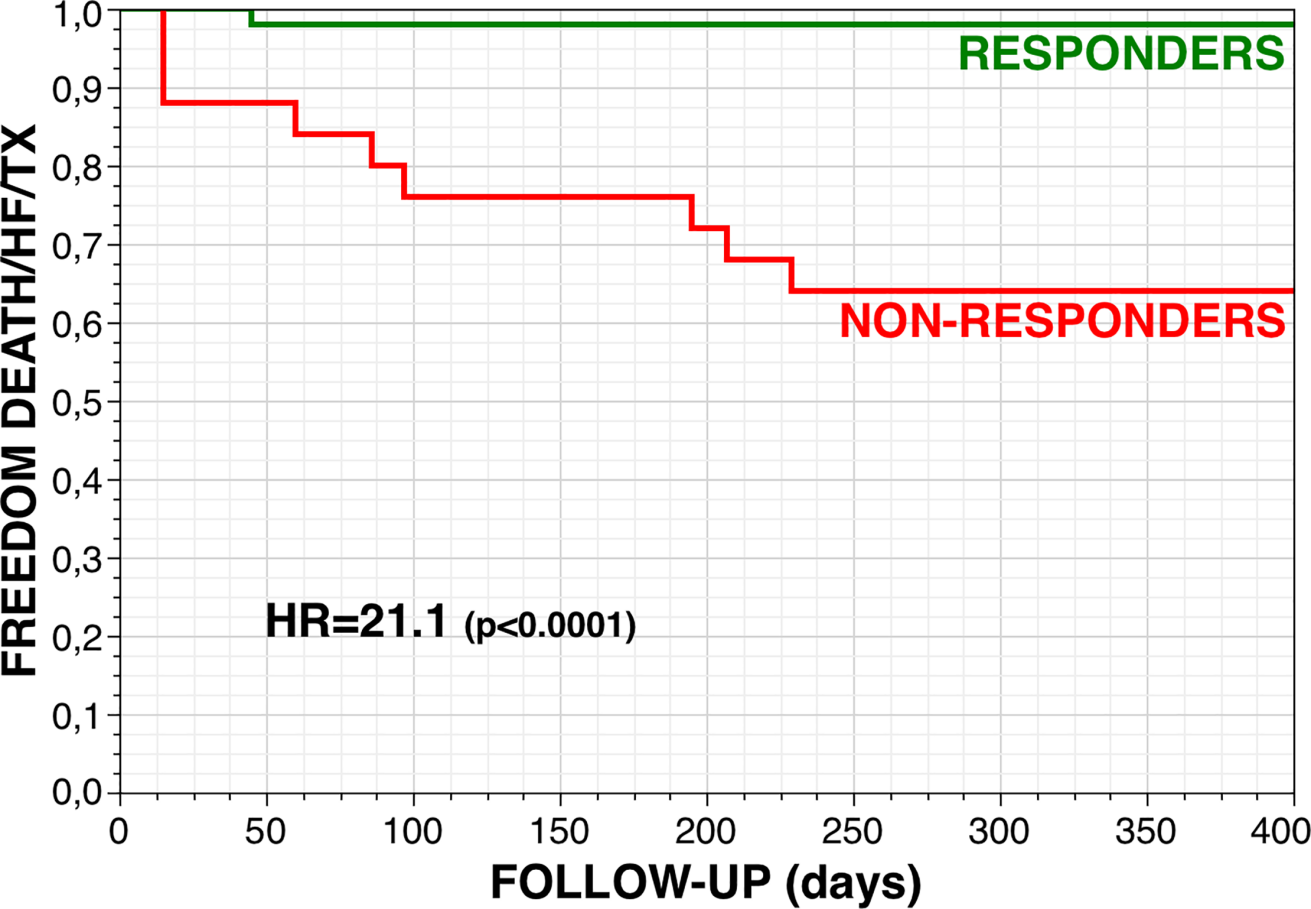
One-year event-free survival (combined end-point: Cardiac death, heart transplantation and/or hospitalization for heart failure) for responders (N = 50, 67%) and non-responders at 6 months.

Only 1 patient in the group of responders was hospitalized for decompensated HF on a cavotricuspid isthmus-dependent right atrial flutter with rapid ventricular rate during the blanking period, and was ablated. Responders at 6 months had a 98% 1-year event-free survival rate with the combined endpoint of cardiac death, heart transplantation and/or HF hospitalization, as compared with a 64% survival in non-responders to ablation (HR 21.1 [3.95–389], p<0.0001) (Fig 1).

### History of cardiomyopathy

**Fig 2:**
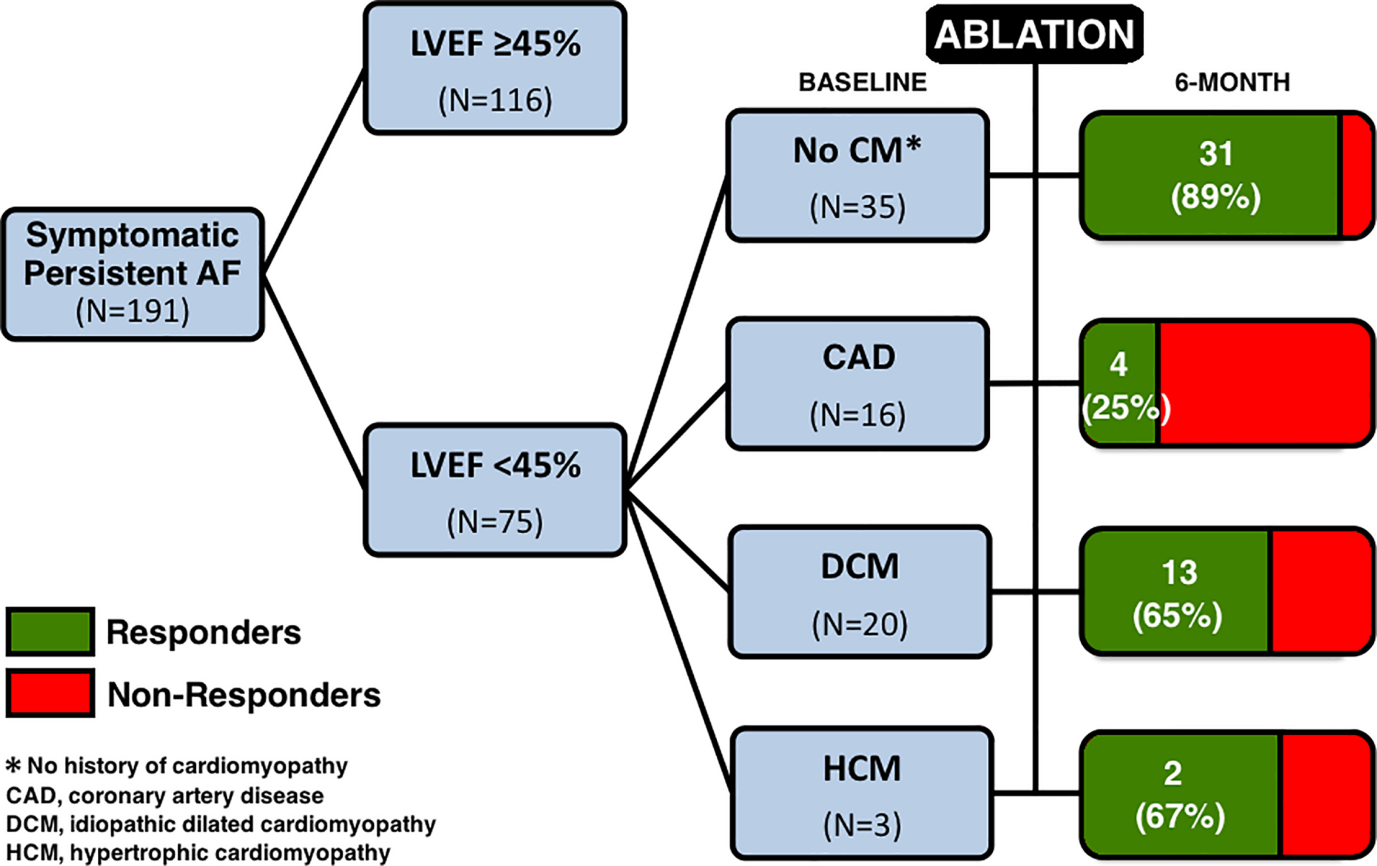
Study flow-chart. Responders to ablation were arbitrarily defined as being those patients fulfilling all the following criteria at 6 months: (1) alive, (2) in sinus rhythm, and (3) with an LVEF ≥50%.

In the subgroup of patients with a history of ischemic heart disease (N = 16), only 4 (25%) were responders, and thus diagnosed with an associated AIC. In the subgroup of patients with an initial diagnosis of idiopathic dilated cardiomyopathy before assessment in our tertiary center (N = 20), 13 (65%) showed complete normalization of LV systolic function after ablation, and whom diagnosis was thus rectified as true AIC (Fig 2).

### Galectin-3

There was a moderate correlation between galectin-3 and BNP levels (R^2^ = 0.32, p<0.0001).

**Fig 3:**
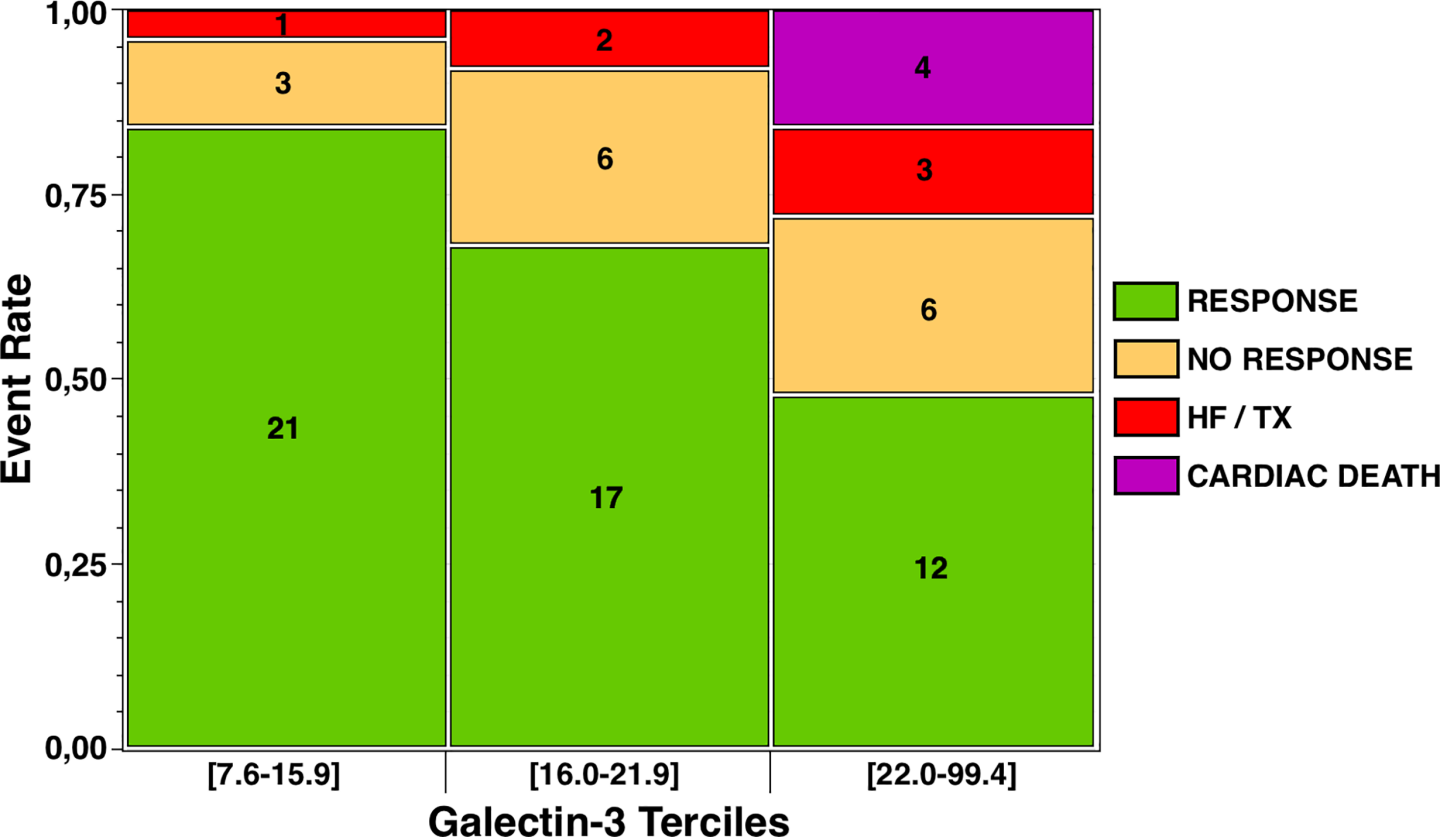
Rates of responders to ablation at 6 months, patients with heart failure hospitalization (HF) or heart transplantation (TX) at 12 months, and those dead from a cardiac cause at 12 months, according to galectin-3 terciles (ng/mL).

Responders had significantly lower galectin-3 levels (Table 1), while non-responders were more likely to be patients in the higher tercile of galectin-3 (p = 0.03) (Fig 3). A galectin-3 level ≥26 ng/mL predicted the absence of response to ablation with a sensitivity of 98%, a specificity of 44%, a positive predictive value of 78%, and a negative predictive value of 92% (area under curve 0.72, p<0.0001). After adjustment through multivariable analyses, galectin-3, not BNP, independently predicted non-response to ablation (adjusted OR 0.89 per unit increase) (Table 2).

**Table 2:** Association between baseline parameters and a response to ablation at 6 months.

	*UNIVARIABLE*		*MULTIVARIABLE*			
			*Model 1*		*Model 2*	
	OR [95% CI]*	*p*	OR [95% CI]*	*p*	OR [95% CI]*	*p*
Age	0.96 [0.91–1.00]	0.08				
Male gender	0.48 [0.10–1.75]	0.28				
Body mass index	1.16 [1.05–1.30]	**0.003**	1.13 [1.01–1.31]	**0.04**	1.14 [1.02–1.31]	**0.03**
Ischemic heart disease	0.09 [0.02–0.32]	**<0.0001**	0.14 [0.03–0.60]	**0.008**	0.14 [0.03–0.59]	**0.007**
Hypertension	0.61 [0.22–1.61]	0.32				
Renal failure	0.32 [0.12–0.88]	**0.03**	1.07 [0.24–5.31]	0.93		
Diabetes mellitus	0.44 [0.15–1.31]	0.14				
Mean heart rate in AF	0.99 [0.96–1.01]	0.36				
LVEF (%)	1.07 [1.00–1.16]	**0.04**	1.01 [0.91–1.12]	0.83		
Left atrial diameter	0.99 [0.83–1.17]	0.87				
BNP	0.91 [0.81–0.98]	**0.02**	0.98 [0.86–1.13]	0.79		
Galectin-3	0.87 [0.79–0.94]	**<0.0001**	0.89 [0.80–0.97]	**0.007**	0.89 [0.80–0.96]	**0.002**

* OR [95% CI], odds ratio with 95% confidence interval. OR value is expressed for continuous variables as per-unit increase for regressor (per-100 units increase for BNP).

**Fig 4:**
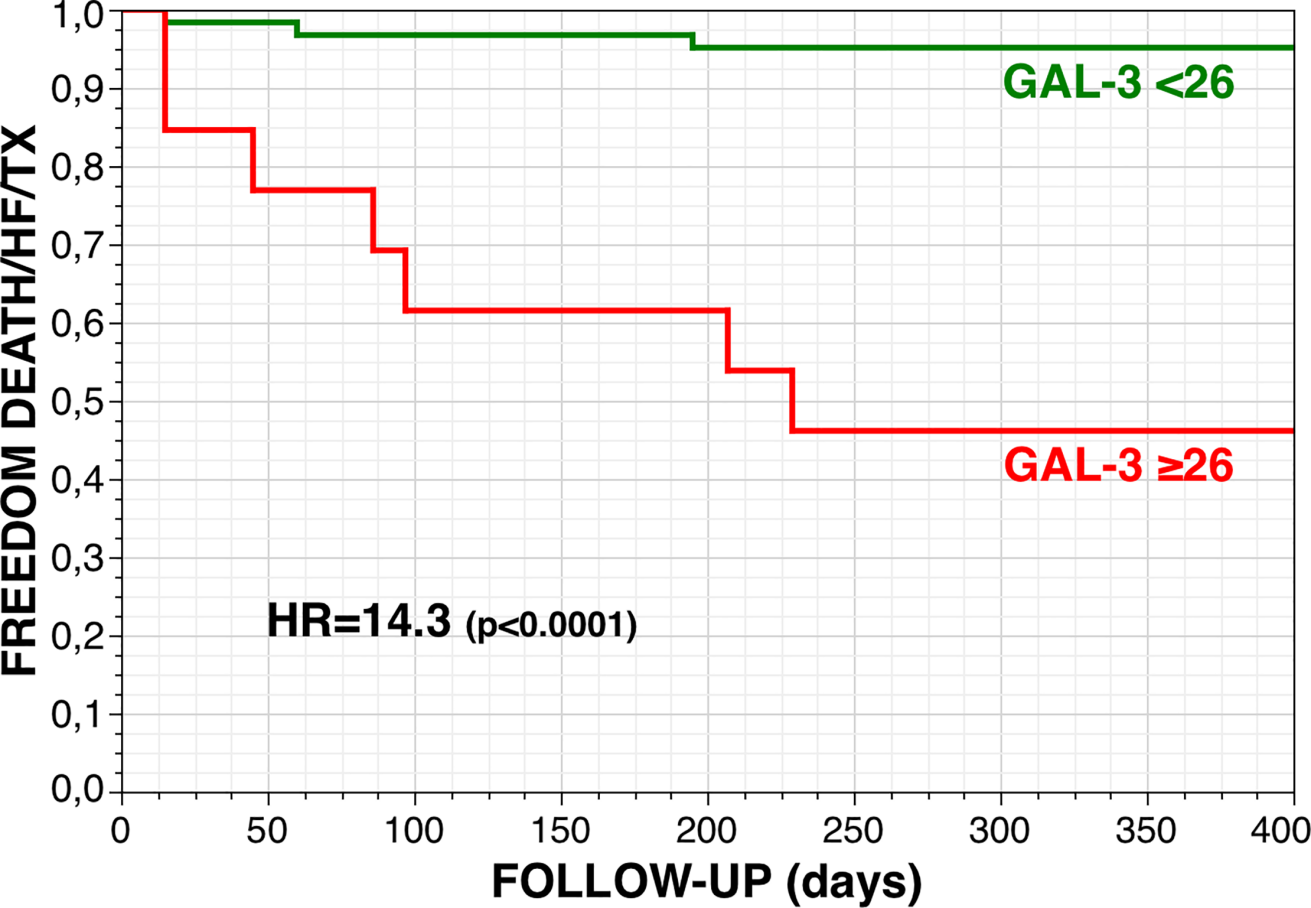
One-year event-free survival (combined end-point: Cardiac death, heart transplantation and/or hospitalization for heart failure) for patients with a baseline galectin-3 level <26 (N = 62, 83%) and ≥26 ng/mL.

Galectin-3 also independently predicted the 1-year combined endpoint of cardiac death, heart transplantation and/or HF hospitalization (unadjusted HR 1.04 [1.02–1.06] per unit increase, p = 0.003). Patients with a galectin-3 baseline level <26 had a 95% 1-year event-free survival rate with the combined endpoint as compared with a 46% survival in patients with a galectin-3 level ≥26 ng/mL (HR 14.3 [3.95–66.5], p<0.0001) (Fig 4).

## Discussion

This cohort study showed that: (1) up to 40% of patients addressed for ablation of persistent AF may present with a reduced left ventricular systolic function; (2) up to 70% of these patients may partially or totally recover left ventricular systolic function after ablation, including some patients with a previous history of structural heart disease; these so-called 'responders to ablation' have a highly favorable 1-year prognosis; (3) higher baseline galectin-3 level identifies non-responders to ablation, i.e. patients at higher risk of arrhythmia recurrence and with a worse outcome.

### Arrhythmia-induced cardiomyopathy

The diagnosis of AIC is retrospective, and can be complex as it may occur in patients either with or without a structural heart disease. In patients presenting with arrhythmia and HF, the arrhythmia itself may be considered secondary and thus not effectively treated. In patients with AF, adequate rate control is specifically mandatory, but rate irregularity may also contribute to an evolution towards AIC [15]. The therapeutic strategy is limited, and eventually involves choosing between strict rhythm control with antiarrhythmic drugs (amiodarone) along with ablation strategies, or strict rate control with pacemaker (or defibrillator) implantation (often biventricular) associated with atrioventricular junction ablation. The randomized PABA-CHF trial randomized 81 patients with symptomatic drug-resistant AF and an LVEF of 40% or less either to a pulmonary vein isolation procedure or to the association of atrioventricular junction ablation and biventricular pacing [16]. At 6 months, patients who had undergone the rhythm control strategy were significantly less symptomatic and had a higher LVEF (35 versus 28%). These results were confirmed in a recent systematic review with a mean 6.1% increase in LVEF following AF ablation in patients with reduced LVEF, which may thus be the preferred strategy in patients with a sufficiently good functional status [17].

We confirm the beneficial effects of persistent AF ablation in the majority of these well-treated patients, even in those with an ischemic cardiomyopathy [18]. Moreover, more than half of all patients were misdiagnosed with a so-called idiopathic dilated cardiomyopathy instead of true AIC. On the other side of the spectrum, for patients with true underlying cardiac disease, prognosis is poor, and the risk of SCD is high (12.5% at 6 months in our study) in the absence of an implantable cardioverter defibrillator [19]. Early identification of patients prone to recover normal LV function is therefore crucial. While patients with a high chance of recovery would require an aggressive rhythm control strategy with ablation, other patients may benefit more from a standard type of HF management, i.e. rate control strategy with prompt implantation of a defibrillator, preferably a biventricular device associated with AV node ablation [20].

### Galectin-3

The prediction of AIC is challenging. Patients with AIC tend to have a smaller LV end-diastolic diameter, but we did not confirm these results after adjustment [21,22]. Lower heart rates in AF do not preclude AIC either. Identification of scar areas using cardiac magnetic resonance with late gadolinium enhancement may also be useful to identify potential responders to ablation [23]. The DECAAF study showed that the extent of atrial fibrosis on MRI, not LA volume, predicted recurrence after AF ablation [3]. Recently, the CAMERA-MRI study showed a significantly better improvement of LV systolic function after catheter ablation in patients with idiopathic cardiomyopathy and an LVEF ≤45% [24]. Interestingly, the study also showed that late gadolinium enhancement negative patients were more likely to normalize LV function (73% versus 21%). However, MRI may not be available for all patients, especially patients already implanted with a cardiac device.

Galectin-3 may be a promising biomarker in that setting for the diagnosis of AF-induced cardiomyopathy. Galectin-3 levels are higher in patients with a structural heart disease, but also in patients with left atrial remodeling [25,26]. In HF patients, higher galectin-3 levels are associated with a higher mortality and risk of developing congestive HF [10,27]. Grandin and colleagues have shown that galectin-3 levels >19.2 ng/ml were associated with a four-fold risk of developing HF [28]. Elevated galectin-3 levels may not only detect cardiac fibrosis, but also contribute to its progression process [29,30]. The fact that fibrosis is involved in both HF and AF progression is of particular interest in the setting of AF-induced cardiomyopathy. Galectin-3 may then identify high-risk patients with either severe HF, or severe atrial disease, or even both. In our study, patients with higher galectin-3 levels (≥17) were at higher risk of non-recovery of LV function after ablation (20 patients, 51%). Patients with even higher levels (≥28) were at very high risk of cardiac death at 6 months (4 patients, 33%), or HF hospitalization at 1 year (7 patients, 58%). The beneficial effects of ablation on outcomes have been recently confirmed in the CASTLE-AF study [31]. Dosage of galectin-3 in patients suffering from AF and a reduced LVEF may help to stratify prognosis and thus to decide on a therapeutic strategy in this population.

### Limitations

Galectin-3 identified so-called "responders to ablation", not patients with an AIC, since some patients with a potential AIC may have had AF recurrence after ablation that prevented them from recovering a normal LV function. The identification of responders may nevertheless represent a more useful clinical tool in this population to select good candidates for ablation. Study of changes of circulating galectin-3 may have been of interest, but serial samples were not collected [32].

## Conclusions

In patients with systolic HF addressed for persistent AF ablation, higher baseline galectin-3 levels identify non-responders to ablation, i.e. patients with a higher risk of arrhythmia recurrence, a lower chance of recovery after ablation (without AIC), and a higher risk of subsequent HF hospitalization and/or cardiac death. This biomarker may be of considerable interest in defining the therapeutic strategy in this population.

## Supporting information

S1 Table Manuscript dataset. (XLSX)

## Abbreviations

AIC: arrhythmia-induced cardiomyopathy
AF: atrial fibrillation
BNP: brain natriuretic peptide
HF: heart failure
LV: left ventricular
LVEF: left ventricular ejection fraction
SCD: sudden cardiac death
